# Evaluation of Serum Immunoglobulins among Individuals Living Near Six Superfund Sites

**DOI:** 10.1289/ehp.8946

**Published:** 2006-03-30

**Authors:** Dhelia M. Williamson, Mary C. White, Charles Poole, David Kleinbaum, Robert Vogt, Kari North

**Affiliations:** 1 Division of Health Studies, Agency for Toxic Substances and Disease Registry, Atlanta, Georgia, USA; 2 Department of Epidemiology, University of North Carolina School of Public Health, Chapel Hill, North Carolina, USA; 3 Centers for Disease Control and Prevention, Atlanta, Georgia, USA; 4 Department of Epidemiology, Emory University, Atlanta, Georgia, USA

**Keywords:** community concerns, environmental exposures, immunoglobulin, Superfund

## Abstract

Residents living in communities near Superfund sites have expressed concern that releases from these facilities affect their health, including adverse effects on their immune systems. We used data from six cross-sectional studies to evaluate whether people who live near several Superfund sites are more likely to have individual immunoglobulin test results (IgA, IgG, and IgM) below or above the reference range than those who live in comparison areas with no Superfund site. Study participants consisted of target-area residents who lived close to a Superfund site and comparison-area residents who were not located near any Superfund or hazardous waste sites. A consistent modeling strategy was used across studies to assess the magnitude of the relationship between area of residence and immunoglobulin test results, adjusting for potential confounders and effect modifiers. In all study areas, the results suggest that people who live near a Superfund site may have been more likely to have IgA test results above the reference range than comparison areas residents regardless of modeling strategy employed. The effect measures were larger for residents who lived in communities near military bases with groundwater contamination. For all analyses the wide confidence intervals reflect uncertainty in the magnitude of these effects. To adequately address the question of whether the immune system is affected by low-level exposures to hazardous substances, we recommend that more functional immunotoxicity tests be conducted in human populations where individual exposure information is available or when it can be reasonably estimated from environmental exposure measurements.

It is estimated that more than 14.5 million people in the contiguous United States live within 1 mi of at least one Superfund site (Heitgerd and Lee 2003). Common contaminants at these sites include heavy metals, volatile organic compounds, and chlorinated hydrocarbons. Many of these substances are present at elevated levels, with the potential for on-site and off-site human contact (Heitgerd and Lee 2003). Residents living in communities near Superfund sites have expressed concern that these releases affect their health, including adverse effects on their immune systems. These concerns have been difficult to address because the available epidemiologic studies regarding immunologic end points are based on occupationally exposed workers or accidentally exposed cohorts exposed to high levels of environmental contaminants (Tryphonas 2001).

To evaluate the potential effect of environmental toxicants on the immune system of residents in communities located near hazardous waste sites, the U.S. Agency for Toxic Substances and Disease Registry (ATSDR) developed an immune test battery for inclusion in community health studies (ATSDR 1994a). This test battery was proposed as a general evaluation of immune status to be used when there was no clear indication of particular health effects or well-defined exposures (Vogt 1991). The basic immune test battery consists of lymphocyte flow cytometric immunophenotyping with specific cluster designation antibody reagents to identify the major types of lymphocytes (CD4 lymphocyte count, CD8 lymphocyte count, CD4:CD8 ratio, T cells, and B cells) and quantitative levels of immunoglobulins (IgA, IgG, and IgM). The tests included in this panel were chosen to assess immune dysfunction in conjunction with self-reported symptoms and illnesses relating to these conditions.

The biomarker panel was applied in several ATSDR cross-sectional health investigations. However, the interpretation of the immunoglobulin values was difficult because of the wide biological variability within populations, nonspecific nature of the tests, and lack of established reference ranges for these tests. In 1998, the Foundation for Blood Research in Scarborough, Maine, provided age- and sex-specific reference limits for IgA, IgG, and IgM (Ritchie et al. 1998). These reference limits were based on automated immunoassay values from 115,017 serum samples, which represents the largest study population in North America obtained within a single laboratory.

The purpose of this study was to reevaluate immunoglobulin levels collected over several investigations by using a consistent approach to data analysis to determine whether individuals who live near several Superfund sites are more likely to have test results below or above the reference range than individuals who live in comparison areas with no Superfund site. Other factors that may be potential confounders or modifiers of these associations are examined as well.

## Materials and Methods

### Study areas

We used data from six cross-sectional studies conducted by the ATSDR between 1991 and 1994 for this analysis. These studies were conducted in Kentucky (ATSDR 1995b), Texas (ATSDR 1995d), California (ATSDR 1996b), Nebraska (ATSDR 1996c), Massachusetts (ATSDR 1998a), and North Carolina (ATSDR 1998b). Table 1 lists the study areas, types of facilities, potential exposure pathways, and contaminants of concern. All six studies were approved by the Centers for Disease Control and Prevention Institutional Review Board. Study participants in all areas gave informed consent before participating.

Four additional cross-sectional studies conducted by ATSDR during this time period were not included in this analysis because they did not include comparison populations (ATSDR 1995a, 1996a) or did not use questionnaires similar to those used in the other studies (ATSDR 1994b, 1995c).

### Study population

Each of the studies included randomly selected community residents. We conducted a census of each community to create the sampling frame. Target area populations consisted of residents living in well-defined areas located close to a Superfund site. Participation rates ranged from 48 to 86% across the six sites and were generally higher among target area residents. We selected each target area based on environmental sampling data that identified contaminated soil, groundwater, surface water, or sediment. Individual exposure data typically were not available. We selected comparison area communities on the basis of demographics and socioeconomic status similar to those of the target area community. Some of the socioeconomic factors considered were style and age of housing, household income, and degree of urbanization. The comparison areas were > 5 miles from the sites of interest and were not located near any other Superfund or hazardous waste sites.

### Data collection

We asked study participants to be interviewed and provide a blood sample. The interview collected information on sociodemographic characteristics (age, race, sex, educational level, years of residence), history of chronic diseases (arthritis, rheumatism, chronic bronchitis, asthma, cancer, multiple sclerosis, lupus), history of specific symptoms (skin rashes, eczema, asthma, bronchitis, allergies), smoking status of the study participant and other household residents, and rating of general health (excellent, good, fair, poor). At three of the study sites (Kentucky, Nebraska, and North Carolina) we collected information about sources of heat for the home (coal stove, fireplace, kerosene or gas heater, or wood stove) and occupational exposures to chemicals (solvents, cleaning agents, dust, insulating materials, paints, gasoline, or kerosene).

### Determination of serum immunoglobulins

Sera were separated by centrifugation at the phlebotomy site and shipped to the Foundation for Blood Research in Scarborough, Maine, where they were refrigerated and assayed within 3 working days. The immunoglobulins measured corresponded to those recommended in the test battery (ATSDR 1994a), which did not include IgE. We tested samples using the immunoturbidimetry method previously described (Hudson et al. 1987). Because of variations among sex and age groups, reference distributions for IgA, IgG, and IgM measurements are sex and age specific (Ritchie et al. 1998).

### Statistical analysis

We used polytomous logistic regression to examine the relationship between area of residence and immunoglobulin test result. We used a three-category classification to code the immunoglobulin test results into those above (> 97.5th percentile), within, or below (< 2.5th percentile) the reference range because increases and decreases of immunoglobulins have been associated with adverse health outcomes (Fischbach 2000). SAS statistical software (version 9.0; SAS Institute Inc., Cary, NC) was used for data management and statistical analysis.

We analyzed the data from each of the six studies using the same modeling strategies to assess the magnitude of the relationship between area of residence and immunoglobulin test results, adjusting for factors that may be potential confounders or modifiers of these associations. The modeling strategies included conducting the following five logistic regression analyses: model 1, no adjustment (crude); model 2, adjustment for sociodemographic variables only; model 3, adjustment for sociodemographic variables and other exposure information (i.e., smoking status of study participant and other household residents; use of coal stove, fireplace, kerosene or gas heater, or wood stove as source of heat for the home; and occupational exposure to chemicals); model 4, adjustment for sociodemographic variables, other exposure information, history of specific symptoms and illness, and rating of general health; and model 5, a backward elimination method described by Kleinbaum (1994). To be included in the regression analysis, studies had to have at least 10 individuals with immunoglobulin test results either below or above the reference range and at least three individuals in each the target and comparison group.

Model 5 included assessing each of the six studies individually using the following strategy. First, interactions between the exposure variable (residence in the target vs. comparison group) and one covariate at a time were examined. Factors that may affect immunologic assay results were considered effect-measure modifiers if the Breslow-Day *p*-value was < 0.5 (Breslow and Day 1980). Next, those covariates not found to be effect-measure modifiers were assessed univariately as potential confounders. A covariate was deemed a confounder if the absolute value of the natural logarithm of the ratio of the unadjusted to adjusted odds ratio (OR) exceeded 0.10. All variables considered effect-measure modifiers or confounders were included in the full model. We assessed each interaction term one at a time using the backward elimination method to eliminate insignificant variables from the model. We assessed significance by comparing the change in log likelihoods (α < 0.20).

## Results

### Descriptive characteristics

Most study participants from each study area were white, were older than 30 years, and had attained at least a high school education (Table 2). The Texas study had a more diverse racial and ethnic composition. Years of residence in the current home varied considerably among the study sites; all of the study participants in Nebraska had lived in their residences for at least 10 years, whereas nearly all study participants in Texas had lived in their residences for < 10 years. Sample sizes of the six studies ranged from 258 participants in the North Carolina study to 912 participants in the Massachusetts study.

Most study participants in both the target and comparison groups for all six areas were in good or excellent health; did not report having a history of specific symptoms, illness, or allergies; did not currently smoke; and did not live in a household in which someone else smoked (Table 3). Only in North Carolina did most study participants report using a coal stove, fireplace, kerosene or gas heater, or wood stove to heat their house. Most study participants in both Kentucky and North Carolina reported having occupational exposure to chemicals.

The proportion of study participants who had individual immunoglobulin test results (IgA, IgG, and IgM) either below or above the reference range varied by specific immunoglobulin and study site (Table 4). Overall, the percentage of study participants in both target and comparison groups with an immunoglobulin test result above the reference range was generally higher than the percentage of study participants with an immunoglobulin test result below the reference range.

### Multivariate analyses

Tables 5–7 show the ORs and 95% confidence intervals (CIs) examining the relationship between area of residence (target vs. comparison) and having an immunoglobulin (IgA, IgG, IgM) test result below or above the reference range in six geographic areas using the different modeling strategies described above. Results from the backward elimination modeling strategy (model 5), which included interaction terms, are discussed individually.

### Immunoglobulin A

#### Results below the reference range

Target area residents in four study areas (California, Kentucky, North Carolina, and Texas) had an increased prevalence of having an IgA test result below the reference range compared with comparison area residents, whereas target area residents in two study areas (Massachusetts and Nebraska) had a decreased prevalence of having an IgA test result below the reference range (Table 5). OR estimates for Texas fell on both sides of the null, depending on which modeling strategy was used. Data were too sparse for North Carolina to generate adjusted OR estimates. All estimates were imprecise [upper-to-lower confidence limit ratio (CLR) > 4] (Poole 2001).

Using the backward elimination strategy (model 5), the models for California and Nebraska included interaction terms. In California, the odds of women living in the target area having an IgA test result below the reference range were 2.66 times those of women living in the comparison area. In contrast, the odds of males living in the target area having an IgA test result below the reference range were 1.16 times those of males living in the comparison area. In Nebraska, individuals who reported having allergies and who lived in the target area were 1.29 times more likely to have an IgA test result below the reference range than those individuals who reported having allergies and who lived in the comparison area. Individuals who reported not having allergies and who lived in the target area in Nebraska were 0.23 times less likely to have an IgA test result below the reference range than those living in the comparison area who did not report having allergies.

#### Results above the reference range

Target area residents in all study areas except North Carolina had an increased prevalence of having IgA test results above the reference range than comparison area residents (data were too sparse for North Carolina to generate OR estimates). Adjusted OR estimates for Texas fell on both sides of the unadjusted estimate, depending on which modeling strategy was used. All estimates were imprecise (CLR > 4).

Using the backward elimination strategy (model 5), two interaction terms were included in the Massachusetts model. The odds of women living in the target area having an IgA test result above the reference range were 11.3 times those of women living in the comparison area, whereas the odds of males living in the target area were 1.66 times those living in the comparison area. For smokers, the odds of having an IgA test result above the reference range were 0.14 times lower among those living in the target area than among those living in the comparison area, whereas nonsmokers were 1.66 times more likely to have an IgA test result above the reference range than nonsmokers living in the comparison area.

### Immunoglobulin G

#### Results below the reference range

Table 6 shows that target area residents in Nebraska and Texas had an increased prevalence of having an IgG test result below the reference range compared with comparison area residents, whereas target area residents in Massachusetts had a decreased prevalence. Data were too sparse in California, Kentucky, and North Carolina to generate OR estimates. Using the backward elimination strategy (model 5), the OR for Texas was substantially lower than the estimates generated from the other models. All estimates were imprecise (CLR ≥ 4).

#### Results above the reference range

When examining the relationship between area of residence and having an IgG test result above the reference range, target area residents in four study areas (Kentucky, Massachusetts, Nebraska, and Texas) had an increased prevalence of IgG test results above the reference range compared with comparison area residents, whereas target area residents in one area (California) had a decreased prevalence. Data were too sparse for North Carolina to generate OR estimates. In Massachusetts, adjustment for additional covariates resulted in the OR estimates falling on the opposite side of the null from the unadjusted OR. In Nebraska, the OR generated from model 4 was approximately the null value. All OR estimates were imprecise (CLR ≥4).

### Immunoglobulin M

#### Results below the reference range

None of the six studies had a total of 10 individuals with IgM test results below the reference range or had at least three individuals in both the target and comparison areas, so no analyses were conducted (Table 7).

#### Results above the reference range

Table 7 shows that target area residents in two study areas (Kentucky and North Carolina) had a modest increased prevalence of having IgM test results above the reference range compared with comparison area residents, whereas target area residents in three study areas (California, Massachusetts, and Texas) had a decreased prevalence. The OR estimates for Nebraska fell on both sides of the null depending on which modeling strategy was used. Data were too sparse for North Carolina to generate adjusted OR estimates. All estimates were imprecise (CLR ≥4).

## Discussion

Evidence from both human and animal studies suggests that a variety of chemicals, including volatile organic compounds and metals, are able to adversely affect the immune system (ATSDR 1994a, 1998b; Burns 1996; National Research Council 1992; Snyder 1994). Xenobiotic toxicants have been shown to either augment the normal immune response, resulting in hypersensitivity, or suppress the immune responses, resulting in immune deficiency. The consequences of immunosuppression may include respiratory infections, opportunistic infections, and cancer (Descotes and Choquet-Kastylevsky 2001). The consequences of immunoenhancement are less well established but include influenza-like reactions such as chills, malaise, and hypotension, as well as exacerbation of chronic infections, psoriasis, Crohn disease, and autoimmune diseases (Descotes and Choquet-Kastylevsky 2001).

Because of the development of standardized reference ranges for IgA, IgG, and IgM, we were able to explore a question that is often raised but rarely investigated: whether individuals living near Superfund sites are more likely to experience changes in immune status than individuals living in areas with no nearby Superfund sites. We examined immunoglobulin test results from six cross-sectional studies conducted in different geographic areas using standardized reference ranges and consistent modeling strategies. Our results suggest that there is variability among the OR estimates generated when examining the relationship between area of residence and having an immunoglobulin test result below or above the reference range. The only consistent pattern observed in all study areas was that individuals who live near a Superfund site were more likely to have IgA test results above the reference range than comparison area residents regardless of modeling strategy employed. However, the wide CI values reflect large uncertainty in the magnitude of these effects.

The effect measures for IgA were consistently larger (OR > 1.5) for residents who lived in communities in Massachusetts and Nebraska near military bases with only groundwater contamination. Although the estimates were imprecise, our results also suggest that individuals living closer to these military bases were less likely to have IgA test results below the reference range than individuals who lived in the comparison neighborhood. In addition, residents who lived near an industrial complex in Kentucky with potential ambient air exposure to heavy metals and other chemicals were more likely to have IgG test results above the reference range than comparison area residents. Because the reference ranges used for this analysis were age and sex adjusted, the observed variability in immunoglobulin results is unlikely to be due to residual confounding by age and sex.

Previous studies have also shown increased IgA levels among individuals living near Superfund sites. ATSDR examined the association between biologic markers of immune system impairment and environmental exposure to cadmium and lead among children and adults living in communities contaminated by mining and smelting operations at four Superfund sites (Sarasua et al. 2000). For children 6–35 months of age, an association was found between increased blood lead levels and increased serum IgA, IgG, and IgM. In adults, urine cadmium was associated with higher levels of IgA after adjustment for age, sex, and other confounders. Additionally, researchers at the University of North Carolina at Chapel Hill examined the effects on the immune system among residents living near the Pesticides Dump Site in Aberdeen, North Carolina, who were potentially exposed to 1,1-dichloro-2,2-bis(*p*-chlorophenyl)ethylene (DDE). The researchers found modestly increased mean IgA levels with increased DDE levels (Vine et al. 2001). Both these studies examined differences in mean IgA levels because standardized reference ranges for immunoglobulins were not available.

The main strength of our study was the unique nature of the data. We used questionnaire and biological data from nearly 4,000 individuals living in six different geographic areas in the United States. The studies used in this analysis were also standardized: they were conducted by the same government agency during a relatively short time period and used the same study design, questionnaire, and immune biomarker test battery. These similarities allowed us to examine patterns of immunoglobulin test results using standardized reference ranges across the different study areas.

A major limitation of this study is the lack of exposure characterization. Individual exposure data were not available, so area of residence was used as a surrogate of exposure. Another limitation was the small number of study participants in some locations, which precluded our ability to have the statistical power to measure stable effect estimates. Also, the information used in this study relied upon self-reported behaviors and risk factors ascertained through interviewer-administered questionnaires. Therefore, there is the possibility of misclassification bias in that people in the target area might have been able to recall symptoms or illnesses to a different extent than people in the comparison areas. This bias could have limited our ability to adjust for these potential confounders in the multivariate models. A final limitation is that serum immunoglobulins are considered a fairly insensitive indicator of immune functions

Because the immune system is a target for adverse effects from exposure to hazardous substances, laboratory tests to measure immune status were included in several ATSDR community health studies. It was thought that the tests would provide quantification of effects given that values outside established reference ranges are generally associated with adverse health outcomes. However, to adequately address the question of whether the immune system is affected by low-level exposures to hazardous substances, we recommend that more functional immunotoxicity tests be conducted in communities located near hazardous waste sites when adequate sample size and individual exposure information are available or when they can be reasonably estimated from environmental exposure measurements. Tests of immune status could be included in such studies but should be tailored for specific types of contaminants or health end points. IgE should be included in the immune status tests because individuals with allergic diseases have been shown to exhibit increased IgE levels whereas decreased levels of IgE are found in cases of autoimmune and other diseases. Information regarding potential confounders should be collected through questionnaires or other mechanisms. Finally, the use of a single reference laboratory would be ideal for quality control and for comparison of results across studies.

## Figures and Tables

**Table 1:** ATSDR cross-sectional studies that included the standardized questionnaire and immune biomarker panel, 1991–1994.

Study location	Type of facility	Exposure pathway	Contaminants
McClellan Air Force Base, Sacramento, CA	Aircraft maintenance facility	Ambient air, sediment, soil, groundwater	Volatile organic compounds, heavy metals
Calvert City Industrial Complex, Calvert City, KY	Manufacturing and handling of chemical compounds	Ambient air	Heavy metals, volatile organic compounds, mineral acids, asbestos, dioxin, radioactive substances, neurotoxic chemicals
Otis Air National Guard, Falmouth, MA	Military training installation	Groundwater	Volatile organic compounds, polychlorinated biphenyls, polycyclic aromatic hydrocarbons
Cornhusker Army Ammunition Plant, Cornhusker, NE	Production of artillery shells, bombs, and rockets	Groundwater	Explosives (RDX and TNT), volatile organic compounds
Caldwell Systems Inc., Caldwell County, NC	Hazardous waste incinerator	Ambient air	Incineration products, phthalates, volatile organic compounds, chromium, arsenic
Brio Refining Co. Inc., Harris County, TX	Regeneration of copper catalysts, recovery of petrochemicals and vinyl chloride	Groundwater	Volatile organic compounds, organic compounds

Abbreviations: RDX, royal demolition explosives (1,3,5-trinitro-1,3,5-triazine); TNT, trinitrotoluene.

**Table 2:** Demographic characteristics of study participants from six cross-sectional studies conducted by ATSDR, 1991–1994 [*n* (%)].

	California (*n* = 655)		Kentucky (*n* = 720)		Massachusetts (*n* = 912)		Nebraska (*n* = 597)		North Carolina (*n* = 258)		Texas (*n* = 774)	
Characteristic	Target (*n* = 453)	Comp (*n* = 202)	Target (*n* = 357)	Comp (*n* = 363)	Target (*n* = 605)	Comp (*n* = 307)	Target (*n* = 297)	Comp (*n* = 300)	Target (*n* = 164)	Comp (*n* = 94)	Target (*n* = 414)	Comp (*n* = 360)
Age												
< 10	11 (2)	8 (4)	11 (3)	12 (3)	20 (3)	10 (3)	0 (0)	0 (0)	0 (0)	0 (0)	17 (4)	17 (5)
10–19	123 (27)	50 (25)	62 (17)	73 (20)	125 (21)	67 (22)	77 (26)	61 (20)	12 (7)	2 (2)	77 (19)	89 (25)
20–29	54 (12)	19 (9)	43 (12)	37 (10)	45 (7)	15 (5)	19 (6)	6 (2)	24 (15)	9 (10)	58 (14)	55 (15)
30–39	64 (14)	36 (18)	65 (18)	70 (19)	118 (20)	59 (19)	12 (4)	21 (7)	20 (12)	12 (13)	147 (36)	128 (36)
40–49	69 (15)	33 (16)	60 (17)	54 (15)	114 (19)	61 (20)	76 (26)	70 (23)	44 (27)	29 (31)	61 (15)	43 (12)
50–59	58 (13)	20 (10)	46 (13)	51 (14)	76 (13)	39 (13)	66 (22)	74 (25)	32 (20)	23 (24)	41 (10)	15 (4)
≥ 60	74 (16)	36 (18)	70 (20)	66 (18)	107 (18)	56 (18)	47 (16)	68 (23)	32 (20)	19 (20)	13 (3)	13 (4)
Sex												
Male	219 (48)	102 (50)	168 (47)	180 (50)	290 (48)	147 (48)	152 (51)	144 (48)	76 (46)	42 (45)	203 (49)	176 (49)
Female	234 (52)	100 (50)	189 (53)	183 (50)	315 (52)	160 (52)	145 (49)	156 (52)	88 (54)	52 (55)	211 (51)	184 (51)
Race												
White	385 (85)	182 (90)	353 (99)	361 (99)	576 (95)	296 (96)	287 (97)	297 (99)	164 (100)	94 (100)	241 (58)	214 (59)
African American	19 (4)	3 (1)	1 (< 1)	0 (0)	8 (1)	2 (1)	1 (< 1)	1 (< 1)	0 (0)	0 (0)	54 (13)	48 (13)
Other	49 (11)	16 (8)	2 (1)	2 (1)	20 (3)	9 (3)	7 (2)	2 (1)	0 (0)	0 (0)	78 (19)	79 (22)
Unknown/missing	0 (0)	1 (< 1)	1 (< 1)	0 (0)	1 (< 1)	0 (0)	2 (< 1)	0 (0)	0 (0)	0 (0)	41 (10)	19 (5)
Hispanic ethnicity												
Yes	67 (15)	30 (15)	5 (1)	5 (1)	10 (2)	2 (1)	3 (1)	10 (3)	0 (0)	0 (0)	65 (16)	81 (23)
No	381 (84)	172 (85)	346 (97)	357 (98)	592 (98)	304 (99)	293 (99)	289 (96)	0 (0)	0 (0)	346 (84)	267 (74)
Missing	5 (1)	0 (0)	6 (2)	1 (< 1)	3 (< 1)	1 (< 1)	1 (< 1)	1 (< 1)	164 (100)	94 (100)	3 (1)	12 (3)
Education												
< High school	205 (45)	79 (39)	107 (30)	121 (33)	154 (25)	75 (24)	81 (27)	75 (25)	60 (37)	25 (27)	115 (28)	133 (37)
High school	109 (24)	55 (27)	128 (36)	128 (35)	289 (48)	149 (49)	122 (41)	106 (35)	45 (27)	31 (33)	200 (48)	166 (46)
> High school	139 (31)	68 (34)	121 (34)	114 (31)	162 (27)	83 (27)	94 (32)	119 (40)	59 (36)	38 (40)	98 (24)	61 (17)
Unknown/missing	0 (0)	0 (0)	1 (< 1)	0 (0)	0 (0)	0 (0)	0 (0)	0 (0)	0 (0)	0 (0)	1 (< 1)	0 (0)
Years in residence												
< 5	102 (23)	95 (47)	117 (33)	136 (37)	74 (12)	32 (10)	0 (0)	0 (0)	26 (16)	10 (11)	252 (61)	210 (58)
5–10	91 (20)	44 (22)	90 (25)	79 (22)	197 (33)	111 (36)	0 (0)	0 (0)	34 (21)	21 (22)	137 (33)	110 (31)
10–19	161 (36)	36 (18)	84 (24)	90 (25)	274 (45)	139 (45)	247 (83)	189 (63)	54 (33)	30 (32)	25 (6)	40 (11)
≥ 20	99 (22)	27 (13)	66 (18)	58 (16)	60 (10)	25 (8)	50 (17)	111 (37)	50 (30)	33 (35)	0 (0)	0 (0)

Comp, comparison group.

**Table 3:** Self-reported symptoms and illnesses, responses to subjective questions, and other exposure information from six cross-sectional studies conducted by ATSDR 1991–1994 [*n* (%)].

	California (*n* = 655)		Kentucky (*n* = 720)		Massachusetts (*n* = 912)		Nebraska (*n* = 597)		North Carolina (*n* = 258)		Texas (*n* = 774)	
Characteristic	Target (*n* = 453)	Comp (*n* = 202)	Target (*n* = 357)	Comp (*n* = 363)	Target (*n* = 605)	Comp (*n* = 307)	Target (*n* = 297)	Comp (*n* = 300)	Target (*n* = 164)	Comp (*n* = 94)	Target (*n* = 414)	Comp (*n* = 360)
Symptoms/Illnesses												
Eczema or skin rashes^a^												
Yes	103 (23)	29 (14)	36 (10)	43 (12)	86 (14)	43 (14)	49 (16)	38 (13)	35 (21)	19 (20)	114 (28)	44 (12)
No	350 (77)	173 (86)	321 (90)	320 (88)	519 (86)	264 (86)	248 (84)	262 (87)	129 (79)	75 (80)	300 (72)	316 (88)
Asthma or bronchitis^a^												
Yes	23 (5)	6 (3)	11 (3)	11 (3)	12 (2)	6 (2)	10 (3)	7 (2)	23 (14)	4 (4)	12 (3)	5 (1)
No	430 (95)	196 (97)	346 (97)	352 (97)	593 (98)	301 (98)	287 (97)	293 (98)	141 (86)	90 (96)	402 (97)	355 (99)
Allergy^c^												
Yes	220 (49)	71 (35)	104 (29)	104 (29)	204 (34)	81 (26)	119 (40)	100 (33)	86 (52)	43 (46)	164 (40)	83 (23)
No	233 (51)	131 (65)	253 (71)	259 (71)	401 (66)	226 (74)	178 (60)	200 (67)	78 (48)	51 (54)	250 (60)	277 (77)
Cancer/immune system^c^												
Yes	61 (13)	22 (11)	36 (10)	43 (12)	86 (14)	43 (14)	49 (16)	38 (13)	41 (25)	22 (23)	35 (8)	14 (4)
No	392 (87)	180 (89)	321 (90)	320 (88)	519 (86)	264 (86)	248 (84)	262 (87)	123 (75)	72 (77)	379 (92)	346 (96)
Other Information												
Overall health												
Excellent	100 (22)	61 (30)	114 (32)	110 (30)	260 (43)	142 (46)	107 (36)	106 (35)	15 (9)	12 (13)	109 (26)	116 (32)
Good	230 (51)	104 (51)	190 (53)	210 (58)	300 (50)	141 (46)	166 (56)	173 (58)	92 (56)	63 (67)	244 (59)	181 (50)
Fair	101 (22)	31 (15)	45 (13)	39 (11)	40 (7)	21 (7)	23 (8)	20 (7)	47 (29)	16 (17)	58 (14)	61 (17)
Poor	22 (5)	6 (3)	7 (2)	4 (1)	4 (< 1)	3 (1)	1 (< 1)	1 (< 1)	10 (6)	3 (3)	2 (< 1)	2 (1)
Missing	0 (0)	0 (0)	1 (< 1)	0 (0)	1 (< 1)	0 (0)	0 (0)	0 (0)	0 (0)	0 (0)	1 (< 1)	0 (0)
Heat home^d^												
Yes	NA	NA	110 (31)	120 (33)	NA	NA	116 (39)	64 (21)	95 (58)	51 (54)	NA	NA
No			247 (69)	243 (67)			181 (61)	236 (79)	69 (42)	43 (46)		
Occupational exposure^e^												
Yes	NA	NA	184 (52)	210 (58)	NA	NA	136 (46)	116 (39)	117 (71)	57 (61)	NA	NA
No			173 (48)	153 (42)			161 (54)	184 (61)	47 (29)	37 (39)		
Currently smoke												
Yes	102 (23)	40 (20)	89 (25)	100 (28)	101 (17)	61 (20)	41 (14)	47 (16)	54 (33)	27 (29)	80 (19)	37 (10)
No	309 (68)	134 (66)	242 (68)	232 (64)	453 (75)	220 (72)	249 (84)	245 (82)	110 (67)	67 (71)	330 (80)	321 (89)
Missing	42 (9)	28 (14)	26 (7)	31 (8)	51 (8)	26 (8)	7 (2)	8 (2)	0 (0)	0 (0)	4 (1)	2 (1)
Someone smoke in house												
Yes	134 (30)	54 (27)	100 (28)	117 (32)	130 (21)	69 (22)	62 (21)	77 (26)	56 (34)	24 (26)	117 (28)	88 (24)
No	277 (61)	120 (59)	230 (64)	215 (59)	424 (70)	213 (69)	228 (77)	215 (72)	108 (66)	70 (74)	295 (71)	269 (75)
Missing	42 (9)	28 (14)	27 (8)	31 (9)	51 (8)	25 (8)	7 (2)	8 (2)	0 (0)	0 (0)	2 (1)	3 (1)

Abbreviations: Comp, comparison group; NA, not applicable.

a Onset since moving to current home.

b Includes allergy, hay fever, watery and burning eyes, irritated nose, severe headaches; onset since moving to current home.

c Includes all cancer types, lupus, osteoarthritis, multiple sclerosis, etc.

d Use of coal stove, fireplace, kerosene or gas heater, or wood stove to heat house.

e Occupational exposures to solvents, cleaning agents, dust, insulating materials, paints, gasoline, or kerosene.

**Table 4:** Serum immunoglobulin results from six cross-sectional studies conducted by ATSDR 1991–1994 [*n* (%)].

	California (*n* = 655)		Kentucky (*n* = 720)		Massachusetts (*n* = 912)		Nebraska (*n* = 597)		North Carolina (*n* = 258)		Texas (*n* = 774)	
Characteristic	Target (*n* = 453)	Comp (*n* = 202)	Target (*n* = 357)	Comp (*n* = 363)	Target (*n* = 605)	Comp (*n* = 307)	Target (*n* = 297)	Comp (*n* = 300)	Target (*n* = 164)	Comp (*n* = 94)	Target (*n* = 414)	Comp (*n* = 360)
IgA												
Below	11 (2)	3 (1)	13 (4)	8 (2)	14 (2)	9 (3)	7 (2)	14 (5)	11 (7)	5 (5)	8 (2)	5 (1)
Within	420 (93)	191 (95)	333 (93)	346 (95)	558 (92)	282 (92)	281 (95)	279 (93)	143 (87)	85 (90)	379 (92)	326 (91)
Above	17 (4)	7 (3)	11 (3)	9 (2)	25 (4)	5 (2)	9 (3)	5 (2)	4 (2)	4 (4)	16 (4)	10 (3)
Missing	5 (1)	1 (< 1)	0 (0)	0 (0)	8 (1)	11 (4)	0 (0)	2 (< 1)	6 (4)	0 (0)	11 (3)	19 (5)
IgG												
Below	2 (< 1)	4 (2)	5 (1)	3 (1)	11 (2)	14 (5)	9 (3)	6 (2)	4 (2)	1 (1)	7 (2)	5 (1)
Within	433 (96)	185 (92)	342 (96)	356 (98)	571 (94)	276 (90)	282 (95)	287 (96)	150 (92)	93 (99)	368 (89)	321 (89)
Above	13 (3)	12 (6)	10 (3)	4 (1)	15 (2)	6 (2)	6 (2)	5 (2)	4 (2)	0 (0)	28 (7)	15 (4)
Missing	5 (1)	1 (< 1)	0 (0)	0 (0)	8 (1)	11 (4)	0 (0)	2 (< 1)	6 (4)	0 (0)	11 (3)	19 (5)
IgM												
Below	1 (< 1)	3 (1)	3 (1)	3 (1)	6 (1)	1 (< 1)	6 (2)	3 (1)	0 (0)	2 (2)	10 (2)	2 (1)
Within	432 (95)	188 (93)	342 (96)	350 (96)	567 (94)	282 (92)	278 (94)	282 (94)	149 (91)	88 (94)	380 (92)	320 (89)
Above	15 (3)	10 (5)	12 (3)	10 (3)	24 (4)	13 (4)	13 (4)	13 (4)	9 (5)	4 (4)	13 (3)	19 (5)
Missing	5 (1)	1 (< 1)	0 (0)	0 (0)	8 (1)	11 (4)	0 (0)	2 (< 1)	6 (4)	0 (0)	11 (3)	19 (5)
One or more												
Below	11 (2)	8 (4)	18 (5)	12 (3)	24 (4)	19 (6)	18 (6)	18 (6)	12 (7)	7 (7)	24 (6)	10 (3)
Within	395 (87)	167 (83)	310 (87)	329 (91)	514 (85)	254 (83)	252 (85)	258 (86)	129 (79)	79 (84)	334 (81)	289 (80)
Above	42 (9)	26 (13)	29 (8)	22 (6)	59 (10)	23 (7)	27 (9)	22 (7)	17 (10)	8 (9)	45 (11)	42 (12)
Missing	5 (1)	1 (< 1)	0 (0)	0 (0)	8 (1)	11 (4)	0 (0)	2 (< 1)	6 (4)	0 (0)	11 (3)	19 (5)

Comp, comparison group.

**Table 5:** OR estimates (95% CIs) for area of residence and IgA test results for six ATSDR studies conducted 1991–1994.

Study area	Model 1^a^	Model 2^b^	Model 3^c^	Model 4^d^	Model 5^e^
Results below the reference range					
California	1.67 (0.46–6.05)	2.05 (0.54–7.88)	1.92 (0.48–7.68)	2.17 (0.52–9.09)	2.66 (0.52–13.51)^f^ 1.16 (0.22–6.17)^g^
Kentucky	1.69 (0.69–4.13)	1.65 (0.67–4.08)	1.27 (0.49–3.28)	1.29 (0.49–3.37)	1.36 (0.54–3.44)
Massachusetts	0.79 (0.34–1.84)	0.80 (0.34–1.88)	0.65 (0.26–1.57)	0.65 (0.26–1.59)	0.63 (0.26–1.52)
Nebraska	0.50 (0.20–1.25)	0.60 (0.23–1.56)	0.58 (0.22–1.57)	0.59 (0.21–1.62)	1.29 (0.06–27.8)^h^ 0.23 (0.05–1.11)^i^
North Carolina	1.31 (0.44–3.89)	[Table-fn tfn10-ehp0114-001065]—	[Table-fn tfn10-ehp0114-001065]—	[Table-fn tfn10-ehp0114-001065]—	[Table-fn tfn10-ehp0114-001065]—
Texas	1.38 (0.45–4.25)	0.98 (0.28–3.44)	1.15 (0.32–4.18)	0.83 (0.20–3.47)	1.16 (0.35–3.88)
Results above the reference range					
California	1.10 (0.45–2.71)	1.35 (0.51–3.62)	1.32 (0.49–3.53)	1.22 (0.45–3.36)	1.17 (0.47–2.91)
Kentucky	1.27 (0.52–3.10)	1.21 (0.49–3.0)	1.13 (0.45–2.84)	1.27 (0.50–3.25)	1.22 (0.49–3.03)
Massachusetts	2.53 (0.96–6.67)	2.46 (0.92–6.54)	2.24 (0.83–6.01)	2.41 (0.88–6.63)	11.29 (0.11–145.53)^f^
					1.66 (0.40–6.83)^g^
					0.14 (0.0–4.52)^j^
					1.66 (0.40–6.83)^k^
Nebraska	1.79 (0.59–5.40)	2.13 (0.58–7.77)	2.02 (0.54–7.61)	1.95 (0.46–8.21)	1.69 (0.56–5.15)
North Carolina	[Table-fn tfn10-ehp0114-001065]—	[Table-fn tfn10-ehp0114-001065]—	[Table-fn tfn10-ehp0114-001065]—	[Table-fn tfn10-ehp0114-001065]—	[Table-fn tfn10-ehp0114-001065]—
Texas	1.38 (0.62–3.07)	1.23 (0.52–2.91)	1.32 (0.56–3.13)	1.71 (0.69–4.23)	1.62 (0.71–3.69)

— , OR estimates could not be calculated.

a Model 1: unadjusted.

b Model 2: adjusted for age, sex, race, Hispanic ethnicity, educational level, years in residence.

c Model 3: adjusted for the model 2 covariates and other exposure information.

d Model 4: adjusted for the model 3 covariates, symptoms, illnesses, and overall health.

e Model 5: adjusted using the backward elimination strategy of Kleinbaum (1994).

f OR for women.

g OR for men.

h OR for individuals with allergies.

i OR for individuals without allergies.

j OR for smokers.

k OR for nonsmokers.

**Table 6:** OR estimates (95% CIs) for area of residence and IgG test results for six ATSDR studies conducted 1991–1994.

Study area	Model 1^a^	Model 2^b^	Model 3^c^	Model 4^d^	Model 5^e^
Results below the reference range					
California	[Table-fn tfn22-ehp0114-001065]—	[Table-fn tfn22-ehp0114-001065]—	[Table-fn tfn22-ehp0114-001065]—	[Table-fn tfn22-ehp0114-001065]—	[Table-fn tfn22-ehp0114-001065]—
Kentucky	[Table-fn tfn22-ehp0114-001065]—	[Table-fn tfn22-ehp0114-001065]—	[Table-fn tfn22-ehp0114-001065]—	[Table-fn tfn22-ehp0114-001065]—	[Table-fn tfn22-ehp0114-001065]—
Massachusetts	0.38 (0.17–0.85)	0.38 (0.17–0.86)	0.40 (0.17–0.94)	0.44 (0.18–1.06)	0.43 (0.18–1.01)
Nebraska	1.53 (0.54–4.34)	1.43 (0.49–4.21)	1.35 (0.44–4.17)	1.31 (0.42–4.13)	1.68 (0.58–4.85)
North Carolina	[Table-fn tfn22-ehp0114-001065]—	[Table-fn tfn22-ehp0114-001065]—	[Table-fn tfn22-ehp0114-001065]—	[Table-fn tfn22-ehp0114-001065]—	[Table-fn tfn22-ehp0114-001065]—
Texas	1.22 (0.38–3.89)	1.83 (0.41–8.08)	1.79 (0.38–8.53)	1.68 (0.27–10.44)	1.03 (0.28–3.80)
Results above the reference range					
California	0.46 (0.21–1.03)	0.38 (0.16–2.13)	0.38 (0.15–0.96)	0.35 (0.14–0.90)	0.39 (0.16–0.94)
Kentucky	2.60 (0.81–8.38)	2.38 (0.70–8.06)	2.39 (0.70–8.16)	2.57 (0.73–9.07)	2.51 (0.78–8.11)
Massachusetts	1.21 (0.46–3.15)	0.92 (0.34–2.50)	0.72 (0.25–2.05)	0.74 (0.25–2.14)	0.90 (0.33–2.44)
Nebraska	1.22 (0.37–4.05)	1.26 (0.33–4.90)	1.36 (0.34–5.50)	1.02 (0.24–4.30)	1.38 (0.41–4.66)
North Carolina	[Table-fn tfn22-ehp0114-001065]—	[Table-fn tfn22-ehp0114-001065]—	[Table-fn tfn22-ehp0114-001065]—	[Table-fn tfn22-ehp0114-001065]—	[Table-fn tfn22-ehp0114-001065]—
Texas	1.63 (0.85–3.10)	1.52 (0.72–3.20)	1.60 (0.75–3.41)	1.52 (0.66–3.49)	1.58 (0.82–3.04)

— , OR estimates could not be calculated.

a Model 1: unadjusted.

b Model 2: adjusted for age, sex, race, Hispanic ethnicity, educational level, years in residence.

c Model 3: adjusted for the model 2 covariates and other exposure information.

d Model 4: adjusted for the model 3 covariates, symptoms, illnesses, and overall health.

e Model 5: adjusted using the backward elimination strategy of Kleinbaum (1994).

**Table 7:** OR estimates (95% CIs) for area of residence and IgM test results for six ATSDR studies conducted 1991–1994.

Study area	Model 1^a^	Model 2^b^	Model 3^c^	Model 4^d^	Model 5^e^
Results below the reference range					
California	[Table-fn tfn28-ehp0114-001065]—	[Table-fn tfn28-ehp0114-001065]—	[Table-fn tfn28-ehp0114-001065]—	[Table-fn tfn28-ehp0114-001065]—	[Table-fn tfn28-ehp0114-001065]—
Kentucky	[Table-fn tfn28-ehp0114-001065]—	[Table-fn tfn28-ehp0114-001065]—	[Table-fn tfn28-ehp0114-001065]—	[Table-fn tfn28-ehp0114-001065]—	[Table-fn tfn28-ehp0114-001065]—
Massachusetts	[Table-fn tfn28-ehp0114-001065]—	[Table-fn tfn28-ehp0114-001065]—	[Table-fn tfn28-ehp0114-001065]—	[Table-fn tfn28-ehp0114-001065]—	[Table-fn tfn28-ehp0114-001065]—
Nebraska	[Table-fn tfn28-ehp0114-001065]—	[Table-fn tfn28-ehp0114-001065]—	[Table-fn tfn28-ehp0114-001065]—	[Table-fn tfn28-ehp0114-001065]—	[Table-fn tfn28-ehp0114-001065]—
North Carolina	[Table-fn tfn28-ehp0114-001065]—	[Table-fn tfn28-ehp0114-001065]—	[Table-fn tfn28-ehp0114-001065]—	[Table-fn tfn28-ehp0114-001065]—	[Table-fn tfn28-ehp0114-001065]—
Texas	[Table-fn tfn28-ehp0114-001065]—	[Table-fn tfn28-ehp0114-001065]—	[Table-fn tfn28-ehp0114-001065]—	[Table-fn tfn28-ehp0114-001065]—	[Table-fn tfn28-ehp0114-001065]—
Results above the reference range					
California	0.65 (0.29–1.48)	0.66 (0.27–1.60)	0.60 (0.24–1.52)	0.60 (0.23–1.56)	0.67 (0.28–1.62)
Kentucky	1.23 (0.52–2.88)	1.21 (0.51–2.90)	1.23 (0.51–2.96)	1.19 (0.48–2.95)	1.18 (0.50–2.79)
Massachusetts	0.92 (0.46–1.83)	0.92 (0.45–1.89)	0.90 (0.44–1.87)	0.85 (0.41–1.76)	0.92 (0.46–1.84)
Nebraska	1.01 (0.46–2.23)	1.22 (0.52–2.84)	1.05 (0.44–2.51)	1.0 (0.42–2.41)	0.98 (0.44–2.17)
North Carolina	1.33 (0.40–4.44)	[Table-fn tfn28-ehp0114-001065]—	[Table-fn tfn28-ehp0114-001065]—	[Table-fn tfn28-ehp0114-001065]—	[Table-fn tfn28-ehp0114-001065]—
Texas	0.58 (0.28–1.19)	0.65 (0.30–1.43)	0.68 (0.31–1.51)	0.58 (0.25–1.38)	0.62 (0.29–1.34)

— , adjusted OR estimates could not be calculated.

a Model 1: unadjusted.

b Model 2: adjusted for age, sex, race, Hispanic ethnicity, educational level, years in residence.

c Model 3: adjusted for the model 2 covariates and other exposure information.

d Model 4: adjusted for the model 3 covariates, symptoms, illnesses, and overall health.

e Model 5: adjusted using the backward elimination strategy of Kleinbaum (1994).
